# P-162. Emergence of ceftriaxone resistant strain in North India

**DOI:** 10.1093/ofid/ofaf695.386

**Published:** 2026-01-11

**Authors:** Bimal Kumar Das, Sushila Dahiya, Sarita Mohapatra, Arti Kapil, Seema Sood

**Affiliations:** All India Institute of Medical Sciences, New Delhi, Delhi, Delhi, India; AIIMS, New Delhi, Delhi, Delhi, India; All India Institute of Medical Sciences, New Delhi, Delhi, Delhi, India; All India Institute of Medical Sciences, New Delhi, Delhi, Delhi, India; All India institute of medical sciences, new delhi, NEW DELHI, Delhi, India

## Abstract

**Background:**

Typhoid fever, a systemic febrile illness caused by Salmonella Typhi (*S.* Typhi) has a complex history of ongoing adaptation and progressive build-up of resistance to commonly used antibiotics.

**Methods:**

All the culture positive enteric fever cases during 2017–2024 presenting to our hospital were included in the study. Antimicrobial susceptibility was done against amoxicillin, chloramphenicol, cotrimoxazole, ciprofloxacin, levofloxacin, ofloxacin, pefloxacin, ceftriaxone and azithromycin as per corresponding CLSI guidelines for each year.

The organism identification and antimicrobial susceptibility was done by standard protocol.

WGS was carried out for molecular characterization of XDR isolate with paired-end 2 x 150 bp reads on Illumina MiSeq (Illumina, USA) employing v2 and v3 chemistry.

**Results:**

Total isolation of blood culture positive typhoidal Salmonella was 596 during the study period (2017-2024). Out of which Salmonella Paratyphi A was 117 followed by 479 Salmonella Typhi.

Pre-COVID 19 - Fluoroquinolones (CIP, LEV, OFL) show notable non susceptibility which is 14–20% followed by 74–89% intermediate susceptibility and exhibiting only 2–6% susceptibility.

While ceftriaxone and cefixime showed complete susceptibility, azithromycin resistance was observed in 2% isolates. Resistance to chloramphenicol, cotrimoxazole and ampicillin (MDR) was recorded in 2-3% isolates.

Post-COVID 19- Fluoroquinolones (CIP, LEV, OFL) shows increase in non-susceptibility which is 28–52% followed by 69–44% intermediate susceptibility and exhibiting only 3–4% susceptibility.

**Conclusion:**

The increase in typhoidal cases suggests a surge in typhoid cases, possibly due to compromised public health systems during and after the pandemic and a shift in its seasonality, is likely to be influenced by environmental and systemic factors. Further epidemiological studies are required for better understanding. The identified XDR strain represents a novel lineage distinct from known XDR isolates in Pakistan. Arrival of XDR *S.* Typhi in Indian subcontinent serves as a warning. The question now is the timeframe for its potential spread to other regions of India.

**Disclosures:**

All Authors: No reported disclosures

